# Gastrin-releasing peptide receptors in the central nervous system: role in brain function and as a drug target

**DOI:** 10.3389/fendo.2012.00159

**Published:** 2012-12-17

**Authors:** Rafael Roesler, Gilberto Schwartsmann

**Affiliations:** ^1^Laboratory of Neuropharmacology and Neural Tumor Biology, Department of Pharmacology, Institute for Basic Health Sciences, Federal University of Rio Grande do SulPorto Alegre, Brazil; ^2^Cancer Research Laboratory, University Hospital Research Center (CPE-HCPA), Federal University of Rio Grande do SulPorto Alegre, Brazil; ^3^National Institute for Translational MedicinePorto Alegre, Brazil; ^4^Department of Internal Medicine, School of Medicine, Federal University of Rio Grande do SulPorto Alegre, Brazil

**Keywords:** gastrin-releasing peptide, gastrin-releasing peptide receptor, bombesin receptors, neuropeptide signaling, brain disorders

## Abstract

Neuropeptides acting on specific cell membrane receptors of the G protein-coupled receptor (GPCR) superfamily regulate a range of important aspects of nervous and neuroendocrine function. Gastrin-releasing peptide (GRP) is a mammalian neuropeptide that binds to the GRP receptor (GRPR, BB2). Increasing evidence indicates that GRPR-mediated signaling in the central nervous system (CNS) plays an important role in regulating brain function, including aspects related to emotional responses, social interaction, memory, and feeding behavior. In addition, some alterations in GRP or GRPR expression or function have been described in patients with neurodegenerative, neurodevelopmental, and psychiatric disorders, as well as in brain tumors. Findings from preclinical models are consistent with the view that the GRPR might play a role in brain disorders, and raise the possibility that GRPR agonists might ameliorate cognitive and social deficits associated with neurological diseases, while antagonists may reduce anxiety and inhibit the growth of some types of brain cancer. Further preclinical and translational studies evaluating the potential therapeutic effects of GRPR ligands are warranted.

## INTRODUCTION

Neuropeptide signaling regulates a variety of aspects of nervous and neuroendocrine function (Hökfelt et al., 2003; Salio et al., 2006). Neuropeptides act by activating specific cell membrane receptors that are members of the G protein-coupled receptor (GPCR) superfamily, leading to stimulation of downstream protein kinase signaling pathways and ultimately altering gene expression (Oh et al., 2006).

Gastrin-releasing peptide (GRP), a neuropeptide originally isolated from the porcine stomach, is a 27-amino acid peptide synthesized as a 148-amino acid precursor (PreproGRP) and subsequently metabolized posttranslationally (Spindel et al., 1984, 1990; Lebacq-Verheyden et al., 1988). GRP is the mammalian homolog of the amphibian 14-amino acid peptide bombesin, isolated from the skin of the European frog *Bombina bombina* in 1970 (Erspamer et al., 1970). GRP and bombesin display similar biological activities and share the same seven C-terminal amino acid sequence. Early experiments examining the effects of bombesin when administered in the brain showed that intracerebroventricular (i.c.v.) infusions of bombesin induced hypothermia and hyperglycemia in rats (Brown et al., 1977a,b). In peripheral tissues, the physiological functions of GRP include regulating gastrin and somatostatin release, gastric acid secretion, pancreatic secretion, gastrointestinal motility, lung development, and chemoattraction in immune system cells (Ruff et al., 1985; Schubert et al., 1991; Del Rio and De la Fuente, 1994; Niebergall-Roth and Singer, 2001; Ohki-Hamazaki et al., 2005; Gonzalez et al., 2008; Jensen et al., 2008b; Czepielewski et al., 2012). Another member of the bombesin-like peptide (BLP) family found in mammals is neuromedin B (NMB), the mammalian equivalent of ranatensin, which acts on the NMB receptor (NMBR; Minamino et al., 1983). An additional peptide originally named neuromedin C (NMC) is in fact a decapeptide of GRP (GRP-10, GRP_18-27_; Minamino et al., 1984). Thus, BLPs in mammalian tissues have been increasingly shown to constitute a class of signaling peptides regulating a large range of physiological functions.

Gastrin-releasing peptide acts by binding to the GRP receptor (GRPR, also called BB2), a GPCR that binds preferentially to GRP and bombesin, with much lower affinity for NMB (Jensen and Gardner, 1981; Moody et al., 1988, 1992; von Schrenck et al., 1989, 1990; Ladenheim et al., 1990, 1992; Wang et al., 1992). Increasing evidence indicates that GRPR-mediated signal transduction in the central nervous system (CNS) plays an important role in regulating behavior, especially aspects related to emotional responses, social interaction, memory, and feeding. In addition, we have proposed that dysfunctions in GRPR expression and signaling might play a role in CNS disorders including anxiety, autism, memory dysfunction associated with neurodegenerative disorders, and brain tumors. Here we review the role of GRPRs in regulating brain function, and its potential as a drug target for CNS disorders.

## MOLECULAR ORGANIZATION OF THE GRPR

All mammalian bombesin receptors (GRPR, NMBR, and the orphan receptor BRS-3 or BB3) exhibit the characteristic seven transmembrane domain structure of GPCRs. This review will focus solely on the GRPR. For a comprehensive review of the classification, nomenclature, structure, expression, signaling, and functions of the different types of bombesin receptors, see Jensen et al. (2008b).

The GRPR, cloned from murine Swiss 3T3 cells in 1990 (Spindel et al., 1990; Battey et al., 1991), is a 384-amino acid protein in humans, mice, and rats. The chromosomal location for the GRPR gene (named *GRPR* in humans and *Grpr* in mice and rats) is at chromosome Xp22.2-p22.13 (human), X F4 (mouse), and Xq21 (rat; Jensen et al., 2008a; **Table 1**).

**Table 1:** Molecular structure of the gastrin-releasing peptide receptor (GRPR).

GRPR (BB2)				
Species	TM	AA	Chromosomal location	Gene name
Human	7	384	Xp22.2-p22.13	*GRPR*
Rat	7	384	Xq21	*Grpr*
Mouse	7	384	X F4	*Grpr*

Aminoacid sequence (*Homo sapiens*)				
(1–60)	MALNDCFLLN	LEVDHFMHCN	ISSHSADLPV	NDDWSHPGIL
	YVIPAVYGVI	ILIGLIGNIT		
(61–120)	LIKIFCTVKS	MRNVPNLFIS	SLALGDLLLL	ITCAPVDASR
	YLADRWLFGR	IGCKLIPFIQ		
(121–180)	LTSVGVSVFT	LTALSADRYK	AIVRPMDIQA	SHALMKICLK
	AAFIWIISML	LAIPEAVFSD		
(181–240)	LHPFHEESTN	QTFISCAPYP	HSNELHPKIH	SMASFLVFYV
	IPLSIISVYY	YFIAKNLIQS		
(241–300)	AYNLPVEGNI	HVKKQIESRK	RLAKTVLVFV	GLFAFCWLPN
	HVIYLYRSYH	YSEVDTSMLH		
(301–360)	FVTSICARLL	AFTNSCVNPF	ALYLLSKSFR	KQFNTQLLCC
	QPGLIIRSHS	TGRSTTCMTS		
(361–384)	LKSTNPSVAT	FSLINGNICH	ERYV	

*Structural data are from Spindel et al. (1990), Battey et al. (1991), Wada et al. (1991), and Jensen et al. (2008a). Modified from Roesler et al. (2012), with permission).*

## GRPR SIGNALING

Experiments using different types of normal and tumor cells from humans and rodents have provided consistent evidence that the GRPR is directly coupled to the G_q_ type of G protein, and GRPR activation leads to an increase in cellular [Ca^2^^+^] and stimulation of the phospholipase C (PLC)/protein kinase C (PKC) and extracellular signal-regulated protein kinase (ERK)/mitogen-activated protein kinase (MAPK) pathways (Hellmich et al., 1999; Chen and Kroog, 2004; Stangelberger et al., 2005). GRPR signaling also interacts with a range of other enzymes (e.g., phospholipases A_2_ and D, tyrosine kinases, phosphatidylinositol 3-kinase – PI3K, and ciclooxigenase-2), growth factor receptor systems (including epidermal growth factor receptor, EGFR, and TrkB), and immediate-early genes (c-fos and c-jun; Szepeshazi et al., 1997; Chatzistamou et al., 2000; Thomas et al., 2005; Hohla et al., 2007; Ishola et al., 2007; Liu et al., 2007; Flores et al., 2008; de Farias et al., 2010; Czepielewski et al., 2012; Petronilho et al., 2012). Data on signaling mechanisms mediating GRPR actions specifically in the CNS will be discussed below.

## GRPR EXPRESSION IN THE CNS

Early studies investigating the presence of bombesin receptors binding sites in the mammalian CNS showed that bombesin could bind with high affinity to rat brain membranes (Moody et al., 1978). Subsequently, autoradiographic studies indicated that areas containing high densities of GRPRs include the olfactory bulb, nucleus accumbens, caudate putamen, central amygdala, dorsal hippocampus, as well as the paraventricular, central medial, and paracentral thalamic nuclei (Wolf et al., 1983; Wolf and Moody, 1985; Zarbin et al., 1985). A detailed immunohistochemical characterization of GRPR expression in the mouse brain showed high GRPR immunoreactivity in the basolateral and central nuclei of the amygdala (BLA and CeA, respectively), hippocampus, hypothalamus, brain stem, nucleus tractus solitarius (NTS), and several cortical areas. Importantly, GRPR expression was restricted to neuronal cell bodies and dendrites, and was not present in axons or glial cells (Kamichi et al., 2005). Thus, the pattern of GRPR location in the brain suggests that it is specifically involved in regulating synaptic transmission. In some rat brain areas, GRPR expression shows marked changes during development – specifically between postnatal (PN) days 1 and 16 – with its expression increasing in the dentate gyrus and decreasing in the caudate putamen and lateral cerebellar nucleus (Wada et al., 1992).

Regarding receptor ligands, the use of radioimmunoassay techniques allowed demonstrating the presence of endogenous BLPs in the rat brain, with high concentrations in brain areas including the NTS, amygdala, and hypothalamus (Moody and Pert, 1979; Moody et al., 1981). GRP mRNA has the highest density in forebrain areas and hypothalamus (Wada et al., 1990; Battey and Wada, 1991; for reviews, see Moody and Merali, 2004; Roesler et al., 2006a; Jensen et al., 2008b).

In the rodent spinal cord, GRPR expression is restricted to lamina I of the dorsal spinal cord, and GRP is expressed in a subset of dorsal root ganglion neurons including lumbar spinothalamic neurons (Sun and Chen, 2007; Fleming et al., 2012; Kozyrev et al., 2012). Importantly, the GRP system in the spinal cord is sexually dimorphic. In male rats, neurons in the L3 and L4 levels of the lumbar spinal cord project to the lower lumbar spinal cord (L5–L6 level) and release GRP onto somatic and autonomic centers containing GRPRs, whereas this system is vestigial in females (Sakamoto et al., 2008; Sakamoto, 2011). This has important implications for the control of male sexual reflexes by GRPR signaling (see below).

## GRPR REGULATION OF CNS FUNCTION

Evidence that GRPRs in the brain and spinal cord regulate several physiological functions has come mostly from *in vivo* studies using pharmacological or genetic manipulation of the GRPR in rats or mice. Below, we summarize relevant findings of selected studies focusing on GRPR regulation of memory, stress and anxiety responses, feeding, itching, and sexual behavior.

### SYNAPTIC PLASTICITY AND MEMORY

In the late 1980s, Flood and Morley (1988) demonstrated that systemic or i.c.v. injections of GRP or bombesin after learning modulated memory retention for a T-maze footshock avoidance task in mice. When i.c.v. infusions were used, both peptides facilitated memory consolidation, whereas systemic injections produced memory enhancement or impairment depending on the drug dose and training conditions. Consistently with these findings, bombesin given after training through systemic injections (Rashidy-Pour and Razvani, 1998) or infusions directly into the NTS (Williams and McGaugh, 1994) enhanced memory retention in rats.

Memory modulation by GRPRs seems to be particularly important for memories involving emotional arousal and fear. Thus, pretraining injections of the GRPR antagonist [Leu13-(psi-CH(2)NH)-Leu14]BN impaired memory for inhibitory avoidance conditioning in mice (Santo-Yamada et al., 2003), and injection of another selective GRPR antagonist, RC-3095, in rats impaired memory for inhibitory avoidance but not for a task with less emotional content, novel object recognition (Roesler et al., 2004b). Similar impairing effects of RC-3095 on inhibitory avoidance memory were obtained with systemic posttraining injections (Roesler et al., 2004c), pre- or posttraining intrahippocampal microinfusions (Roesler et al., 2003; Venturella et al., 2005; Dantas et al., 2006; Preissler et al., 2007), or posttraining infusions into the BLA (Roesler et al., 2004c). The effects of the GRPR antagonist followed a typical inverted U-shaped dose–response pattern, in which intermediate doses resulted in memory impairment, whereas higher doses had no effect or produced memory enhancement (Roesler et al., 2003, 2004b; Dantas et al., 2006). Conversely, intrahippocampal infusion of bombesin resulted in enhancement of inhibitory avoidance memory at intermediate doses and impairment at higher doses (Roesler et al., 2006b). In addition to influencing memory formation, pharmacological manipulation GRPRs in specific brain areas has been shown to regulate fear memory expression, extinction, and reconsolidation-like processes (Luft et al., 2006, 2008; Mountney et al., 2006, 2008; Merali et al., 2011). For example, infusion of the GRPR antagonist RC-3095 into the rat dorsal hippocampus after memory reactivation blocks the extinction and reconsolidation of fear memory (Luft et al., 2006, 2008; for a review, see Roesler et al., 2012).

The role of GRPRs in regulating fear memory and synaptic plasticity has also been revealed by genetic studies using GRPR knockout mice. Contextual and cued fear conditioning were enhanced by the genetic deletion of GRPR, whereas spatial in the Morris water maze was unaffected. The enhancement of fear memory in GRPR knockout mice was accompanied by enhanced synaptic plasticity measured by long-term potentiation (LTP) in the amygdala. In wild-type mice, GRPR was preferentially expressed in amygdalar inhibitory interneurons releasing gamma-aminobutyric acid (GABA), and GRP might be released as a co-transmitter from glutamatergic neurons to activate preferentially GRPRs located on GABAergic interneurons to stimulate inhibitory transmission within the amygdala and function as an inhibitory constraint for the formation of fear-motivated memories (Shumyatsky et al., 2002).

Additional studies recently found enhanced retention and impaired extinction of cued fear conditioning, associated with an increase in c-fos activity in the BLA and reduced c-fos in the prefrontal cortex, in GRPR knockout mice. However, these mice showed unaltered contextual fear conditioning, multiple-trial cued fear conditioning, and conditioned taste aversion (Chaperon et al., 2012; Martel et al., 2012). Together, these findings indicate that the GRPR acts as a negative regulator of synaptic plasticity in the BLA and specific types of fear conditioning. However, the use of first generation knockout mouse models might confound the interpretation of the results, given that they do not allow the investigation of separate phases of memory (encoding, consolidation, and expression), and knockout mice might have up-regulation of compensatory pathways and non-specific alterations in CNS development in response to gene ablation (reviewed in Roesler et al., 2012).

**FIGURE 1:**
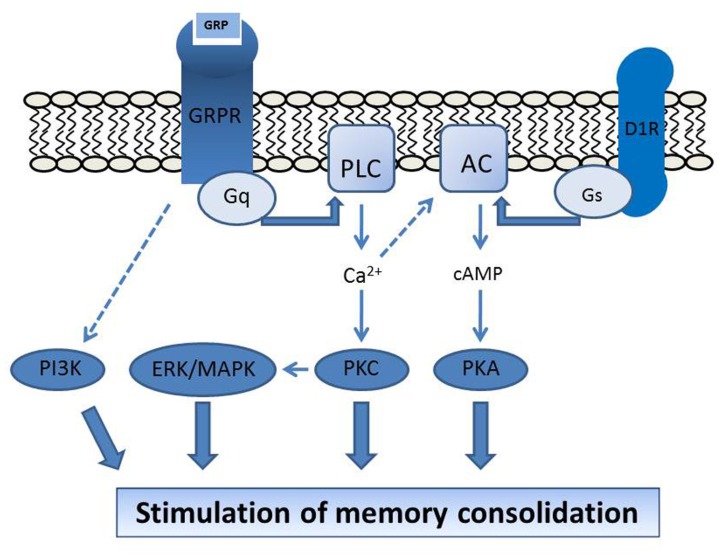
**Proposed molecular mechanisms mediating GRPR regulation of brain function.** The stimulation of hippocampal memory consolidation by GRPR activation depends on PKC, MAPK, PKA, and PI3K, and is potentiated by activation of the D1R/cAMP/PKA pathway (Roesler et al., 2006a, 2009, 2012). GRPR activation at the postsynaptic membrane is coupled to G_q_ protein activity and increases in [Ca^2^^+^], leading to stimulation of the PLC/PKC and ERK/MAPK pathways. D1R is coupled to the G_s_ protein and adenylyl cyclase (AC) activation. The D1R-induced cAMP signal might be potentiated by [Ca^2^^+^]-induced stimulation of [Ca^2^^+^]-responsive types of AC (Wong et al., 1999; Chan and Wong, 2005; Roesler et al., 2006b, 2012), providing a possible mechanism for the requirement of cAMP/PKA signaling for GRPR influences on memory. Modified from Roesler et al. (2006b), 2012, with permission.

We have shown that a number of signal transduction mechanisms downstream of receptor activation are involved in mediating memory regulation by the GRPR. In the CA1 area of the dorsal hippocampus, memory enhancement induced by bombesin was prevented by inhibitors of PKC, MAPK, PKA, and PI3K (Roesler et al., 2006b, 2009, 2012), and potentiated by coinfusion of stimulators of the dopamine D1/D5 receptor (D1R)/cAMP/PKA pathway, namely the D1R agonist SKF 38393, the adenylyl cyclase activator forskolin, and the cAMP analog 8-Br-cAMP (Roesler et al., 2006b). These findings indicate that the PKC, MAPK, PI3K, and PKA pathways are critical in mediating memory modulation by hippocampal GRPRs, and that GRPR activation can interact with cAMP/PKA signaling in enhancing hippocampal memory formation (**Figure 1**). GRPRs in the rat brain also show functional interactions with other growth factor systems including basic fibroblast growth factor (bFGF/FGF-2), nerve growth factor (NGF), and brain-derived neurotrophic factor (BDNF; Kauer-Sant’Anna et al., 2007; Preissler et al., 2007).

### EMOTIONAL BEHAVIOR

Gastrin-releasing peptide and GRPR are highly expressed in brain regions, such as the amygdala, activated by stressful stimuli, and, as discussed above, GRPR signaling is likely to be a major regulator of memory associated with fear and emotional arousal. Merali and colleagues have shown that chronic stressor exposure leads to an elevation of GRP levels in the anterior pituitary in rats, and GRP release in the rat amygdala is increased in response to exposure to a shock. GRP may stimulate the release of adrenocorticotropic hormone (ACTH), playing a role in mediating the corticotropin-releasing hormone (CRH) stress response, and increasing the activity of the hypothalamic–pituitary–adrenal (HPA) axis. In addition, bombesin administration induces endocrine, autonomic, and behavioral effects associated with stress, and bombesin receptor antagonists attenuate the behavioral and neurochemical effects of stressors (Merali et al., 2002, 2009; Moody and Merali, 2004; Mountney et al., 2011). Moreover, we have shown that systemic administration of a GRPR antagonist can induce an anxiogenic-like effect in the elevated plus maze test in rats (Martins et al., 2005). Together, these data suggest that brain GRPRs might regulate emotional behavioral and responses to stress.

### FEEDING BEHAVIOR

It has been known for over 30 years that systemic or i.c.v. administration of bombesin or GRP in rats reduces the intake of liquid and solid food in rats (Gibbs et al., 1981). Similar effects on meal size are observed after systemic bombesin injections in mice and intravenous (i.v.) injections in baboons and humans (Gibbs, 1985). In addition, brief vena caval infusions of GRP and NMB in rats, given alone or together at the onset of the first nocturnal meal, significantly reduced meal size and duration (Rushing et al., 1996), and bombesin or GRP given systemically extended the duration of the intermeal interval (Thaw et al., 1998). The suppression of glucose intake induced by systemic administration of GRP or bombesin was blocked by infusion of a GRPR antagonist into the fourth ventricle in rats (Ladenheim et al., 1996), and was absent in GRPR knockout mice (Hampton et al., 1998; Ladenheim et al., 2002), indicating that central GRPRs are critical in mediating the effects of peripheral bombesin and GRP on feeding. In addition, GRPR knockout mice ate significantly more at each meal than wild-type controls (although total 24 h food consumption was equivalent), and showed elevated body weight compared with wild-type littermates beginning at 45 weeks of age (Ladenheim et al., 2002). The finding that systemic GRP potently reduced independent intake of both sucrose and milk from a bottle but did not affect intraoral intake of either solution indicated that the GRPR regulates the appetitive-related aspects of the feeding process (Rushing and Houpt, 1999). The amygdala is likely a key brain area involved in mediating the regulatory action of GRPRs on feeding: bilateral infusion of GRP into the central amygdala produced a transient inhibition of food intake, an effect that was prevented by the GRPR antagonist [Leu(13)-psi(CH(2)NH)-Leu(14)]BN (Fekete et al., 2002).

These findings provide strong support for a role of GRP/GRPR signaling in regulating feeding. It has been proposed that BLPs may also be released from the gastrointestinal tract in response to food ingestion, acting to bridge the gut and brain to inhibit further food intake. Conversely, the suppression of release of BLPs in the brain may trigger the initiation of a feeding episode (reviewed in Merali et al., 1999).

### SEXUAL BEHAVIOR

One of the most exciting recent developments in GRPR research was the identification by Sakamoto et al. (2008) of a sexually dimorphic GRPR system in the spinal cord that is crucial in regulating male sexual function. In male rats, but not in females or males with a dysfunctional androgen receptor gene, GRP-containing neurons in the upper lumbar spinal cord innervate lower lumbar regions controlling erection and ejaculation. Pharmacological stimulation of spinal GRP receptors restores penile reflexes and ejaculation after castration, whereas intrathecal administration of the GRPR antagonist RC-3095 inhibits penile reflexes and ejaculations. The inhibitory effect of castration on GRP expression in this spinal center suggests that androgen signaling plays a major role in regulating GRP expression in the male spinal cord (Sakamoto et al., 2009b). Moreover, exposure to traumatic stress decreases the local GRP content and reduces penile reflexes in male rats (Sakamoto et al., 2009a; Sakamoto, 2010). Thus, GRP/GRPR signaling has emerged as a new target for the understanding of psychogenic erectile dysfunction and the development of potential therapeutic approaches to masculine reproductive dysfunction (Sakamoto et al., 2008, 2009a,b; Sakamoto and Kawata, 2009; Sakamoto, 2010, 2011).

### ITCHING

Another function in which GRPRs in the spinal cord have been shown to play a major role is itching. GRPR knockout mice show normal thermal, mechanical, inflammatory, and pain responses, but reduced responses to pruritogenic stimuli, and GRP-induced pruritus in wild-type mice is blocked by intrathecal administration of a GRPR antagonist (Sun and Chen, 2007). The selective ablation of GRPR-expressing lamina I neurons in the mouse spinal cord of mice results in scratching deficits in response to itching stimuli, but does not affect pain behaviors (Sun et al., 2009). These findings allowed the identification of GRPR as a central molecular mediator of the itch sensation in the spinal cord (Sun and Chen, 2007; Sun et al., 2009).

A recent seminal study showed that the μ-opioid receptor (MOR) isoform MOR1D heterodimerizes with GRPR in the spinal cord to relay itch information. Blocking the association between MOR1D and GRPR attenuates morphine-induced scratching. Morphine triggers internalization of both GRPR and MOR1D, whereas GRP specifically triggers both GRPR internalization and morphine-independent scratching. These data suggest that opioid-induced itch is independent of opioid analgesia and occurs via cross-activation of GRPR signaling by MOR1D heterodimerization (Liu et al., 2011).

## POSSIBLE ROLE OF ALTERATIONS IN GRPR EXPRESSION AND SIGNALING IN THE PATHOGENESIS OF BRAIN DISORDERS

Since GRPRs are highly expressed in neurons in brain areas including the hippocampus and BLA, and regulate crucial aspects of behavior that can be altered in patients with CNS disorders, it is possible that deregulated GRPR signaling contribute to the pathogenesis of neurological and psychiatric diseases. Although a causative role of GRPR dysfunction in CNS disorders has not been directly established, some alterations in the levels of BLPs peptides or GRPR density or function have been observed in patients with psychiatric, neurodegenerative, and neurodevelopmental disorders. In addition, the use of preclinical models has provided further evidence indicating a role for the GRPR in some CNS pathologies. Based on these findings, we have put forward that the GRPR may be a novel molecular target for the development of therapeutic strategies for patients with neurological and psychiatric disorders (Roesler et al., 2004a, 2006a). **Table 2** summarizes the findings from studies examining possible alterations in GRP and GRPR content or signaling found in patients with brain disorders.

**Table 2:** Findings from selected studies examining possible alterations in the GRPR system in patients with CNS disorders. Modified from Roesler et al. (2006a), with permission.

CNS disorder	Main findings	Reference
Parkinson’s disease	Reduced levels of BLPs peptides in caudate nucleus and globus pallidus	Bissette et al. (1985)
Parkinson’s disease	Normal bombesin-like immunoreactivity in adrenal medullary tissue	Stoddard et al. (1991)
Alzheimer’s disease	Reduced bombesin receptor density and enhanced bombesin-induced calcium release in fibroblasts	Ito et al. (1994)
Alzheimer’s disease	Reduced bombesin-induced calcium mobilization in fibrobasts	Gibson et al. (1997)
Autism	X;8 translocation in the GRPR gene	Ishikawa-Brush et al. (1997)
Autism	No association with two polymorphic sites in the second exon of the GRPR gene	Marui et al. (2004)
Autism	C6S and L181F mutations in the GRPR gene	Seidita et al. (2008)
Schizophrenia	Reduced radioimmunoassay-detectable bombesin in the CSF	Gerner et al. (1985)
Schizophrenia	Reduced urinary levels of BLPs	Olincy et al. (1999)
Anxiety disorders	No association between GRP and GRPR genes and panic disorders	Hodges et al. (2009)
Eating disorders	Reduced GRP levels in the CSF of women who were recovered from bulimia nervosa	Frank et al. (2001)
Brain tumors	GRPR overexpression in glioma	Flores et al. (2010)

### NEURODEGENERATIVE DISORDERS

The concentration of BLPs was found to be significantly reduced in the caudate nucleus and globus pallidus of patients with Parkinson’s disease (PD; Bissette et al., 1985). However, Stoddard et al. (1991) found no alterations in bombesin-like immunoreactivity in the adrenal medullary tissue of patients with PD, although the concentration of several other neuropeptides was reduced. A reduction in bombesin receptor density and altered bombesin-induced calcium signaling have been reported in fibroblasts from patients with Alzheimer’s disease (AD; Ito et al., 1994; Gibson et al., 1997). For example, in fibroblasts from patients with familial AD presenting the Swedish APP670/671 mutation, elevations in calcium induced by bombesin were reduced by 40% (Gibson et al., 1997).

**FIGURE 2:**
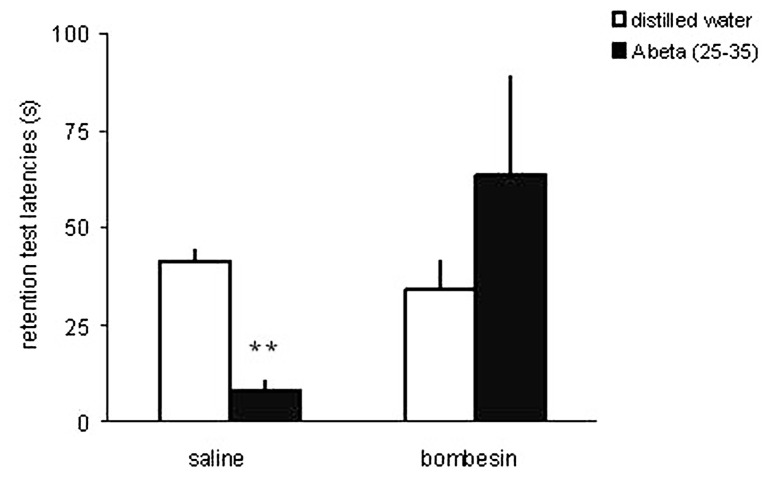
**The GRPR agonist bombesin prevents memory impairment induced by beta-amyloid peptide in the rat hippocampus.** Data are mean ± SEM retention test step-down latencies (s), in an inhibitory avoidance conditioning, of rats given a bilateral infusion of the GRPR agonist bombesin (BB; 0.002 μg) or saline (SAL; control group) 10 min before being trained in IA, and beta-amyloid peptide (Abeta; 25–35) or distilled water (DW; controls) immediately after IA training. The number of animals was 8–14 per group. **P < 0.01 compared to the control group treated with SAL and DW. Reproduced from Roesler et al. (2006b), with permission.

Using the memory impairment produced by a microinfusion of a low dose of beta-amyloid peptide (25–35; Abeta) into the rat CA1 area of the dorsal hippocampus as a model of memory dysfunction associated with AD, we showed that an intrahippocampal infusion of bombesin completely prevented the Abeta-induced impairment in inhibitory avoidance memory (Roesler et al., 2006b; **Figure 2**). This finding provided preliminary preclinical evidence suggesting that pharmacological stimulation of the GRPR might rescue memory deficits associated with AD.

### NEURODEVELOPMENTAL DISORDERS

The first evidence suggesting that the GRPR might be a candidate gene in autism spectrum disorders (ASD) was the finding of a translocation breakpoint on the X chromosome in the first intron of the *GRPR* gene in a patient with autism accompanied by mental retardation and epilepsy (Ishikawa-Brush et al., 1997). Although a subsequent study investigating two polymorphic sites in the second exon of the *GRPR* gene in patients did not support the *GRPR* as a candidate locus for autism (Marui et al., 2004), more recently a possible role of C6S and L181F mutations of the *GRPR* gene in GRPR function and ASD was found in two patients (Seidita et al., 2008).

**FIGURE 3:**
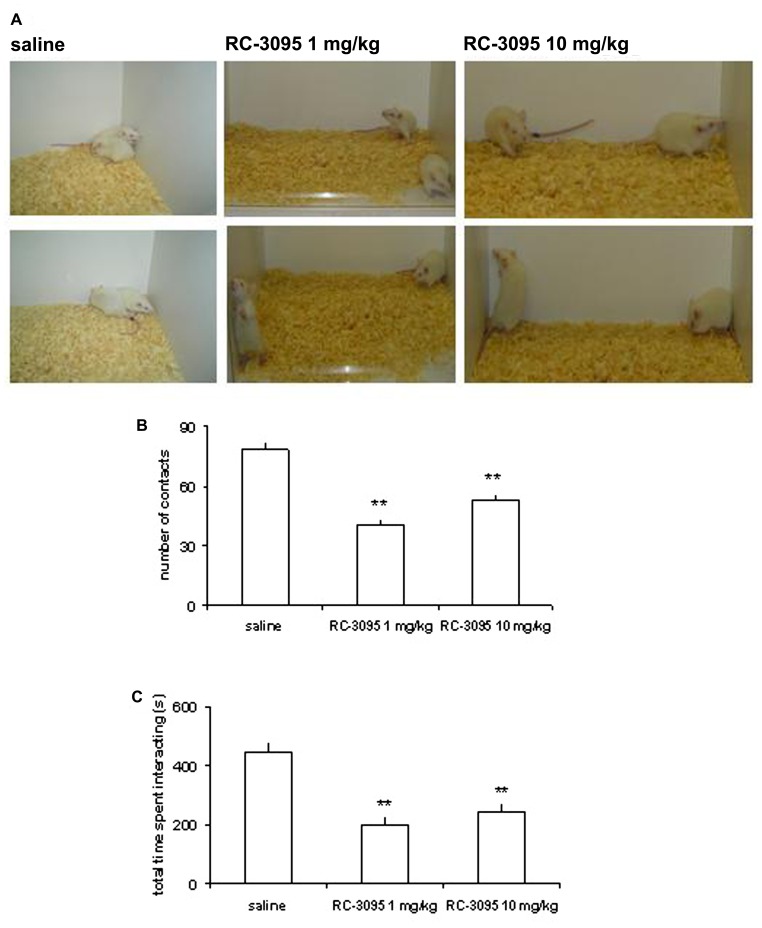
**GRPR blockade during CNS development in rats results in long-lasting behavioral alterations associated with experimental models of autistic spectrum disorders (ASDs).** Rats were given intraperitoneal injections of saline (SAL; control group) or the GRPR antagonist RC-3095 (1 or 10 mg/kg) twice daily from postnatal days (PN) 1 to 10. A social behavior test was carried out at PN 30. **(A)** Representative photographs of rats given SAL or RC-3095 (1 or 10 mg/kg) during the social interaction test. **(B)** Mean ± SEM number of social contacts. **(C)** Mean ± SEM time spent engaged in social interaction (in seconds). The number of animals was 6–7 per group; **P < 0.01 compared to the control group. Reproduced from Presti-Torres et al. (2007), with permission.

In order to examine the role of GRPR in CNS development and its possible involvement in ASD, we submitted rat pups to pharmacological GRPR blockade by systemic administration of RC-3095 from PN days 1–10, and examined long-lasting behavioral and molecular alterations produced by this treatment. Rats given neonatal RC-3095 showed pronounced deficits in social interaction (a hallmark of rodent models of ASD) when tested at PN days 30–35 (Presti-Torres et al., 2007; **Figure 3**) or PN day 60 (Presti-Torres et al., 2012). In addition, RC-3095-treated rats showed impaired 24-h retention of memory for inhibitory avoidance and novel object recognition, whereas body weight during development, open field behavior, and short-term memory were not affected (Presti-Torres et al., 2007, 2012). Neonatal GRPR blockade also reduced maternal odor preference, a behavioral measure of attachment behavior (Garcia et al., 2010). The impairment in social behavior induced by GRPR blockade was rescued by treatment with the atypical antipsychotic clozapine (Presti-Torres et al., 2012). Together, these findings suggest that GRPR blockade during CNS development can lead to specific behavioral alterations that are consistent with ASD, and support the possibility that abnormal GRPR expression or function during development might play a role in disease pathogenesis. Also, we have proposed that neonatal GRPR blockade in rats may serve as a novel animal model of ASD (Presti-Torres et al., 2007, 2012).

### OTHER NEUROPSYCHIATRIC DISORDERS

The findings from rodent studies discussed above, indicating that normal GRPR function during development might be important for behaviors related to social interaction, attachment, and cognition, and that clozapine rescues social behavior deficits produced by GRPR blockade, are also consistent with the possibility that GRPR signaling is altered in schizophrenia. In addition, we found that GRPR blockade by systemic injections of RC-3095 prevent apomorphine-induced stereotypy in mice and amphetamine-induced hyperlocomotion in rats, which are models of schizophrenic psychosis and mania (Meller et al., 2004; Kauer-Sant’Anna et al., 2007). In patients with schizophrenia, a reduction in the levels of radioimmunoassay-detectable bombesin in the cerebrospinal fluid (CSF; Gerner et al., 1985), and reduced urinary levels of BLPs (Olincy et al., 1999) have been found. Further studies using samples from patients and animal models are required to examine whether GRPR signaling is involved in schizophrenia.

As reviewed above, data from animal studies also consistently show that GRPRs in brain areas including the amygdala regulate memory related to fear and anxiety responses, raising the possibility that GRPR signaling plays a role in anxiety disorders (Moody and Merali, 2004; Roesler et al., 2012). For example, pharmacological manipulation of the GRPR in the hippocampus can affect extinction and reconsolidation of fear memory, which are preclinical models used in the investigation and screening of potential therapeutic strategies for post-traumatic stress disorder (PTSD) and other fear-related disorders (Luft et al., 2006, 2008). In postmortem analyses of brains from suicides compared to control subjects, Merali et al. (2006) reported discrete alterations in the levels of GRP and NMB. More recently, however, the possibility that GRP and GRPR are candidate genes in panic disorders was not confirmed in an association and linkage analysis (Hodges et al., 2009).

Anxiety disorders may show comorbidity with eating disorders, anorexia and bulimia nervosa. Given the important role of GRPR in regulating feeding behavior (see above), it is possible that it contributes to eating disorders. One study found significantly reduced GRP levels in the CSF of women who were recovered from bulimia nervosa, compared to women recovered from anorexia or healthy control women. The authors suggested that persistent alterations in GRP levels after recovery indicate that this alteration might be trait-related and contribute to episodic hyperphagia in patients with bulimia nervosa (Frank et al., 2001).

### BRAIN TUMORS

**FIGURE 4:**
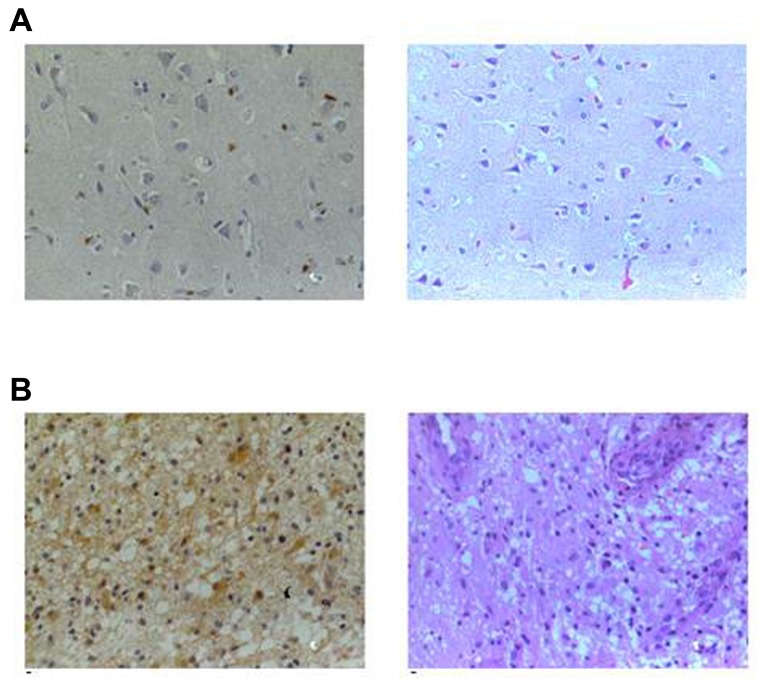
**GRPR content in human normal brain tissue and brain tumors.** Representative sections of **(A)** normal brain and **(B)** astrocytoma grade IV from an immunohistochemical study of GRPR content from samples of patients with gliomas and normal brain samples. GRPR staining is shown in the right column (brown, ×400) and hematoxylin–eosin (HE) in the left column (×400). GRPR staining in the normal brain tissue is restricted to neuronal bodies and dendrites, whereas its presence in astrocytoma samples is widespread. Sections were incubated with anti-GRPR antibody, sequentially treated with biotinylated anti-rabbit IgG and streptavidin-biotin peroxidase solution, and then developed with diaminobenzidine as chromogen. Modified from Flores et al. (2010), with permission.

Gastrin-releasing peptide receptor overexpression has been demonstrated in many types of cancer (Cornelio et al., 2007), and we have recently shown widespread expression and a high content of GRPR in human glioma, the most common and lethal type of neurological cancer (Flores et al., 2010; **Figure 4**). GRPR activation by GRP or bombesin stimulates the growth of glioma cell lines (Moody et al., 1989; Pinski et al., 1994; Sharif et al., 1997; de Farias et al., 2008; Flores et al., 2008). We have recently shown that the stimulatory effect of GRPR activation on proliferation of glioma cells depends on PI3K signaling (Flores et al., 2008) and is potentiated by co-activation of the cAMP/PKA pathway (de Farias et al., 2008; reviewed in Roesler et al., 2010).

**FIGURE 5:**
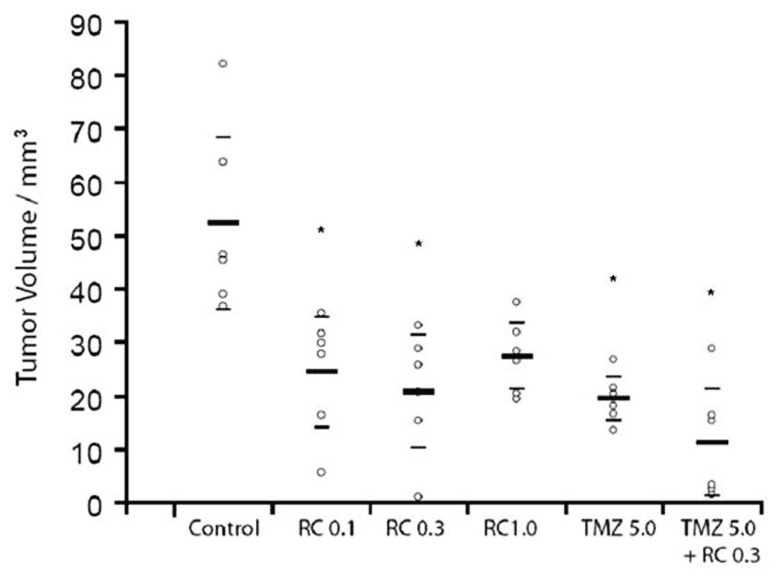
**A GRPR antagonist inhibits the growth of experimental brain tumors.** Rats implanted with C6 experimental gliomas in the striatum were treated for seven consecutive days with intraperitoneal injections of the GRPR antagonist RC-3095 alone (0.1, 0.3, and 1.0 mg/kg twice a day), temozolomide (TMZ) alone (5 mg/kg once a day), or RC-3095 combined with TMZ. Control animals were injected with vehicle. Pharmacological treatments were initiated 10 days after tumor implantation. The number of animals was 6 rats per group. Tumor size was measured 20 days after tumor implantation. Data are shown as median (interquartile ranges) tumor volume (mm^3^). Values for individual animals are shown by dots; *P < 0.002 compared to control animals. Reproduced from de Oliveira et al. (2009), with permission.

Gastrin-releasing peptide receptor antagonists inhibit the growth of human U-87MG and U-373MG gliomas xenografted into nude mice (Pinski et al., 1994; Kiaris et al., 1999). In addition, GRPR antagonism by RC-3095, alone or combined with temozolomide, significantly reduced the growth of C6 gliomas both *in vitro* and *in vivo*, with the combined administration of TMZ and RC-3095 being the most effective treatment (**Figure 5**; de Oliveira et al., 2009). These findings strongly suggest that targeting GRPR may be a promising strategy for the development of novel therapies against glioma. The GRPR might also regulate the growth of neuroblastoma (Kim et al., 2002; Qiao et al., 2008; Abujamra et al., 2009), although, in contrast, we could not find a role for GRPR in regulating the *in vitro* growth of medulloblastoma, the most common brain cancer of childhood (Schmidt et al., 2009).

## GRPR LIGANDS AS CANDIDATE THERAPEUTIC DRUGS IN BRAIN DISORDERS

The evidence reviewed above indicates that the GRPR might be considered a novel molecular target in different types of CNS disorders, and raise the possibility that GRPR agonists might ameliorate cognitive and social deficits associated with neurological diseases, while antagonists may, for example, reduce anxiety and inhibit the growth of some types of brain cancer. Studies examining the effects of GRP administration on satiety and eating behavior in humans (Gutzwiller et al., 1994), as well as a phase I trial of the GRPR antagonist RC-3095 in patients with solid tumors (Schwartsmann et al., 2006) have suggested that GRP and peptidergic GRPR antagonists can be safely administered intravenously in human subjects. Thus, the potential therapeutic effect of GRPR ligands in preclinical models as well as in patients with CNS disorders warrants further investigation.

## Conflict of Interest Statement

The authors declare that the research was conducted in the absence of any commercial or financial relationships that could be construed as a potential conflict of interest.
